# Sex-Based Differences in Gastroduodenal Artery Morphometry

**DOI:** 10.7759/cureus.116666

**Published:** 2026-09-21

**Authors:** Morgan W Stewart, Jenna M Watts, Vincent J Schmidt, Simran Aulakh, Cameron T Smith, Forrest Dunivin, Julia Kirkland, Melissa Zolnierz

**Affiliations:** 1 Surgery, Kansas City University, Joplin, USA; 2 Internal Medicine, Kansas City University, Joplin, USA; 3 Primary Care, Kansas City University, Joplin, USA; 4 Pain Management, Kansas City University, Kansas City, USA; 5 Pathology and Anatomical Sciences, Kansas City University, Joplin, USA; 6 Osteopathic Medicine, Kansas City University, Joplin, USA; 7 Anatomical Sciences, Kansas City University, Joplin, USA; 8 Anatomy, Kansas City University, Joplin, USA

**Keywords:** computed tomography angiography, gastroduodenal artery, morphometric analysis, sex differences, vascular anatomy

## Abstract

Introduction: The gastroduodenal artery (GDA) supplies the upper gastrointestinal tract, and variation in its geometry may influence endovascular access and hepatobiliary interventions. This study evaluated sex-based differences in the GDA branching angle and diameter using standardized morphometric analysis.

Methods: This retrospective cadaveric morphometric study included 28 formalin-embalmed donors (12 males and 16 females) examined between 2024 and 2025. Inclusion criteria required an intact GDA and common hepatic artery suitable for analysis. Following targeted vessel dissection, perpendicular images were obtained with in-plane calibration and analyzed using ImageJ (National Institutes of Health, Bethesda, MD, USA). Branching angle and mid-segment diameter were measured independently by two blinded observers. Sex-based differences were assessed using independent-samples *t*-tests, applying Welch's correction when the assumption of homogeneity of variance was violated.

Results: Twenty-eight donors (mean age 77 ± 8 years; 12 males and 16 females) were evaluated. Male donors demonstrated significantly smaller GDA branching angles than female donors (95.83° ± 23.79° vs. 119.51° ± 17.75°; *p* = 0.006) and significantly larger diameters (5.67 ± 2.57 mm vs. 3.17 ± 0.70 mm; *p* = 0.007).

Conclusion: Sex-based differences were identified in GDA geometry, which may influence computed tomography (CT) angiography interpretation and interventional planning.

## Introduction

Variation in gastroduodenal artery (GDA) geometry may influence vascular access and procedural complexity during endovascular interventions and hepatopancreatobiliary surgery [1-8]. Despite its clinical relevance, quantitative characterization of GDA morphology remains limited, particularly regarding features that affect catheter navigation, such as branching angle and vessel diameter. Whether sex-based differences contribute to variation in GDA geometry has not been systematically evaluated.

Previous studies have described variation within the celiac axis and hepatic arterial system, but focused morphometric analyses of the GDA are scarce [9,10]. Experimental and computational studies demonstrated that arterial geometry influences flow dynamics, wall shear stress, and pressure distribution, with smaller branching angles associated with increased hemodynamic stress [2,11-14]. These geometric characteristics may also affect technical aspects of endovascular procedures, including catheter stability and vascular access.

This cadaveric morphometric study aimed to determine whether GDA branching angle and diameter differ between male and female donors using standardized image-based measurements. These measurements were intended to characterize sex-based anatomical variation in the GDA, with potential relevance to computed tomography (CT) angiography interpretation and interventional planning.

This work was previously presented at the Kansas City University Research Symposium, held April 3-5, 2025.

## Materials and methods

Donors

This retrospective cadaveric morphometric study was approved by the Institutional Review Board (Kansas City University Institutional Biosafety Committee; protocol #2148042). The study complied with all relevant institutional and ethical guidelines for cadaveric research, and informed consent was waived.

A total of 52 formalin-embalmed donors were identified through the institutional anatomical donor program between 2024 and 2025. Inclusion criteria required an intact GDA and common hepatic artery (CHA) suitable for morphometric analysis. Donors were excluded if the GDA was damaged during dissection or if prior regional surgery altered vascular anatomy. After application of these criteria, 28 donors (12 males and 16 females) were included in the final analysis. Donor sex was obtained from institutional records.

Dissection protocol

Standardized dissections were initially performed by first-year medical students according to Grant’s Dissector [15] under faculty supervision. The investigators then performed targeted dissections to expose the origin of the GDA from the CHA and its proximal course.

All included donors demonstrated a GDA originating from the CHA. No additional traction was applied to the vessels beyond routine exposure to minimize geometric distortion. The lead investigator verified anatomical landmarks and vessel identification before imaging acquisition.

Imaging acquisition

Images were obtained using a tablet-based camera (iPad, 10th generation; Apple Inc., Cupertino, CA, USA) mounted on a tripod positioned perpendicular to the dissection plane at a fixed height of 30 cm to minimize parallax error. Anatomical landmarks, including the CHA, stomach, and pancreas, were aligned within the field of view (Figure 1).

**Figure 1:**
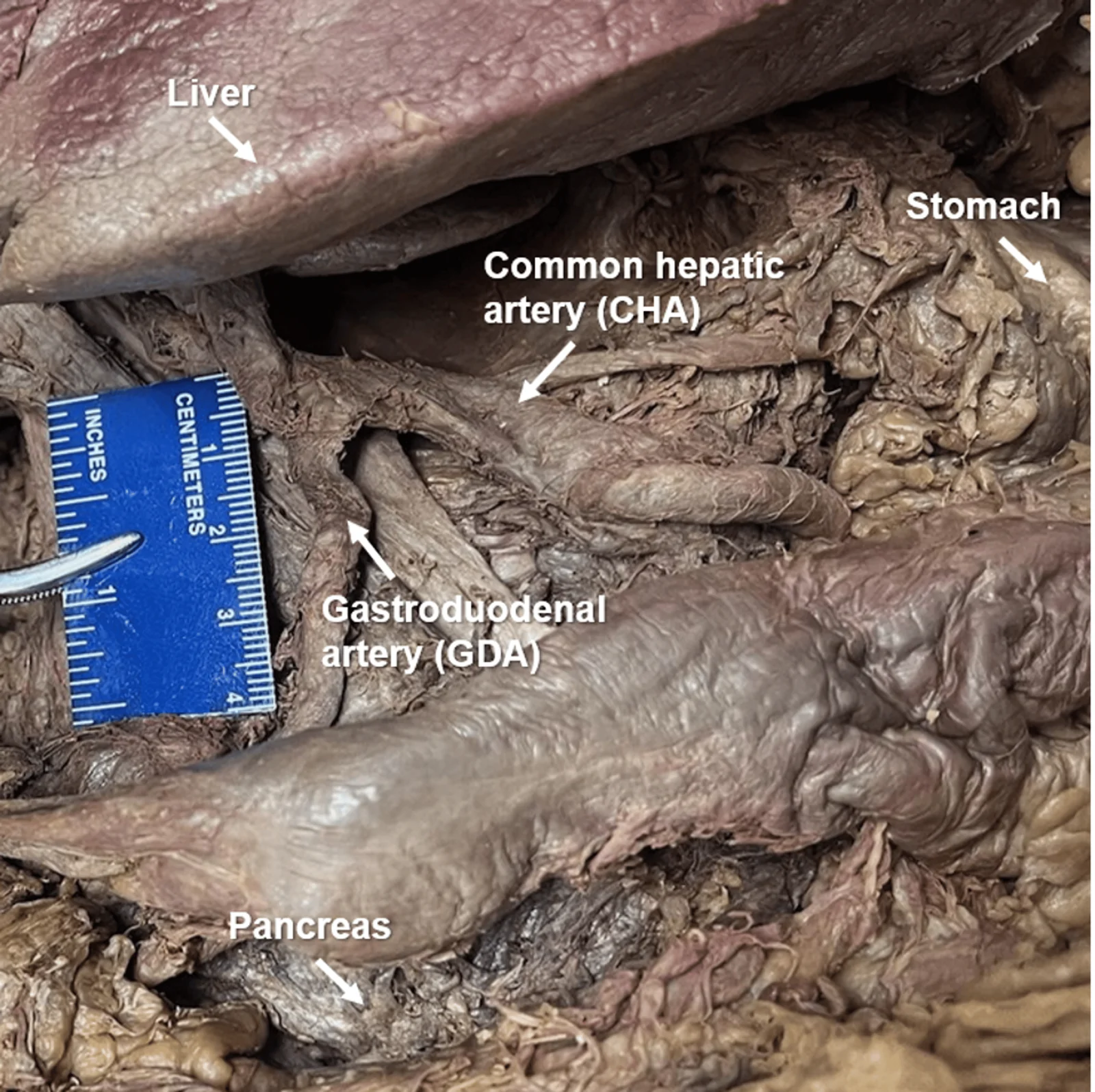
Dissection field demonstrating the origin of the gastroduodenal artery (GDA) from the common hepatic artery (CHA) Labeled structures include the liver, stomach, pancreas, GDA, and CHA. Arrows indicate structure location. A metric ruler positioned within the imaging plane is shown for scale.

A metric ruler positioned in the same plane as the GDA was used for calibration and minimization of projection error. For diameter assessment, the vessel segment was positioned distal side up on a support tray while maintaining the same camera-specimen distance and perpendicular orientation used for branching angle imaging (Figure 2). One image was acquired for branching angle analysis, and one for diameter analysis per donor.

**Figure 2:**
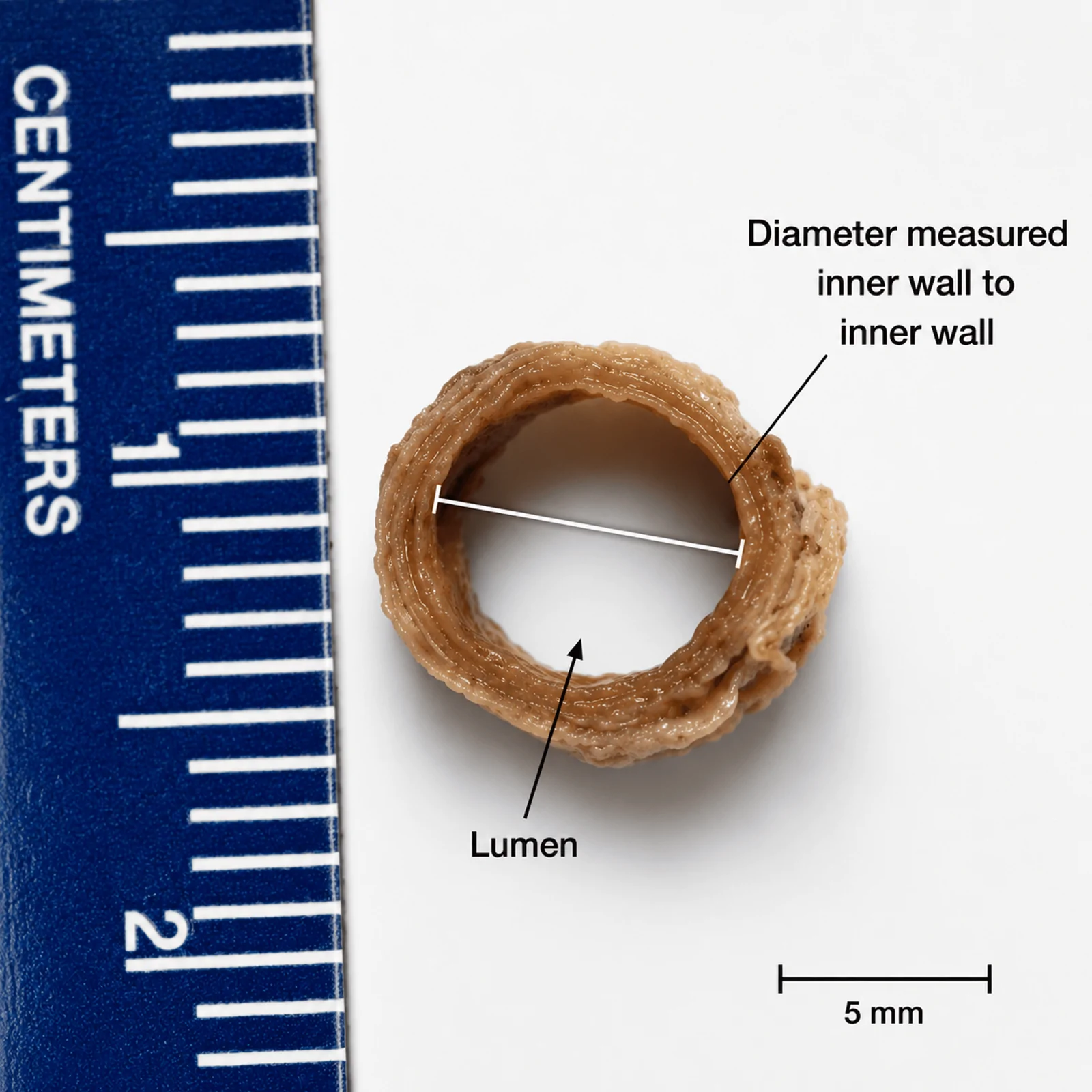
Cross-sectional image of the gastroduodenal artery (GDA) used for morphometric diameter assessment Diameter was measured perpendicular to the vessel axis from inner wall to inner wall (white line). The arrow indicates the lumen. A metric ruler and 5 mm scale bar are shown for reference.

Morphometric measurements

GDA diameter and branching angle were measured using ImageJ (version 1.54p; National Institutes of Health, Bethesda, MD, USA) [16]. Diameter was measured perpendicular to the vessel axis (inner wall to inner wall) at the midpoint of the GDA segment between its origin at the CHA and the first branch encountered.

The branching angle was defined as the angle between the centerline vectors of the CHA and the GDA at their junction. Measurements were obtained in the plane that best represented the visible anatomical course of both vessels at the branching site, with the imaging plane selected consistently across specimens based on vessel alignment at the GDA origin.

Two trained observers independently performed all measurements according to a predefined protocol and were blinded to donor sex. Each observer obtained two measurements of branching angle and diameter for each donor, resulting in four measurements per variable per donor. Measurements were reviewed for consistency, with anatomical landmarks and measurement points re-evaluated when necessary. No measurements required exclusion following review. The median of the four measurements was used as the final value for statistical analysis.

Statistical analysis

Statistical analyses were performed using SPSS (version 29; IBM Corp., Armonk, NY, USA). Continuous variables were expressed as mean ± standard deviation. Normality was assessed using the Shapiro-Wilk test, and homogeneity of variances was assessed using Levene’s test.

Differences between male and female donors were assessed using independent-samples *t*-tests. Welch’s *t*-test was used when the assumption of homogeneity of variance was violated. For variables that departed from normality, the Mann-Whitney U test was also performed as a sensitivity analysis to assess the robustness of the findings. Effect sizes were calculated as Cohen’s *d* using the pooled standard deviation. Mean between-group differences were reported with 95% confidence intervals (CIs). A two-sided *p* < 0.05 was considered statistically significant.

## Results

Study cohort

Twenty-eight donors (mean age 77 ± 8 years; 12 males and 16 females) met the inclusion criteria and were included in the final analysis (Table 1).

**Table 1:** Demographic characteristics of the study cohort Values are presented as mean, mean ± standard deviation, or n (%), as indicated.

Characteristic	Male	Female	Total
Mean age (years)	75	79	77 ± 8
Donors, n (%)	12 (42.9)	16 (57.1)	28 (100.0)

Branching angle

The mean GDA branching angle was smaller in male donors than in female donors (95.83° ± 23.79 vs. 119.51° ± 17.75; Figure 3, Table 2). Branching angles ranged from 56.68° to 147.60° in male donors and from 86.38° to 156.88° in female donors.

**Figure 3:**
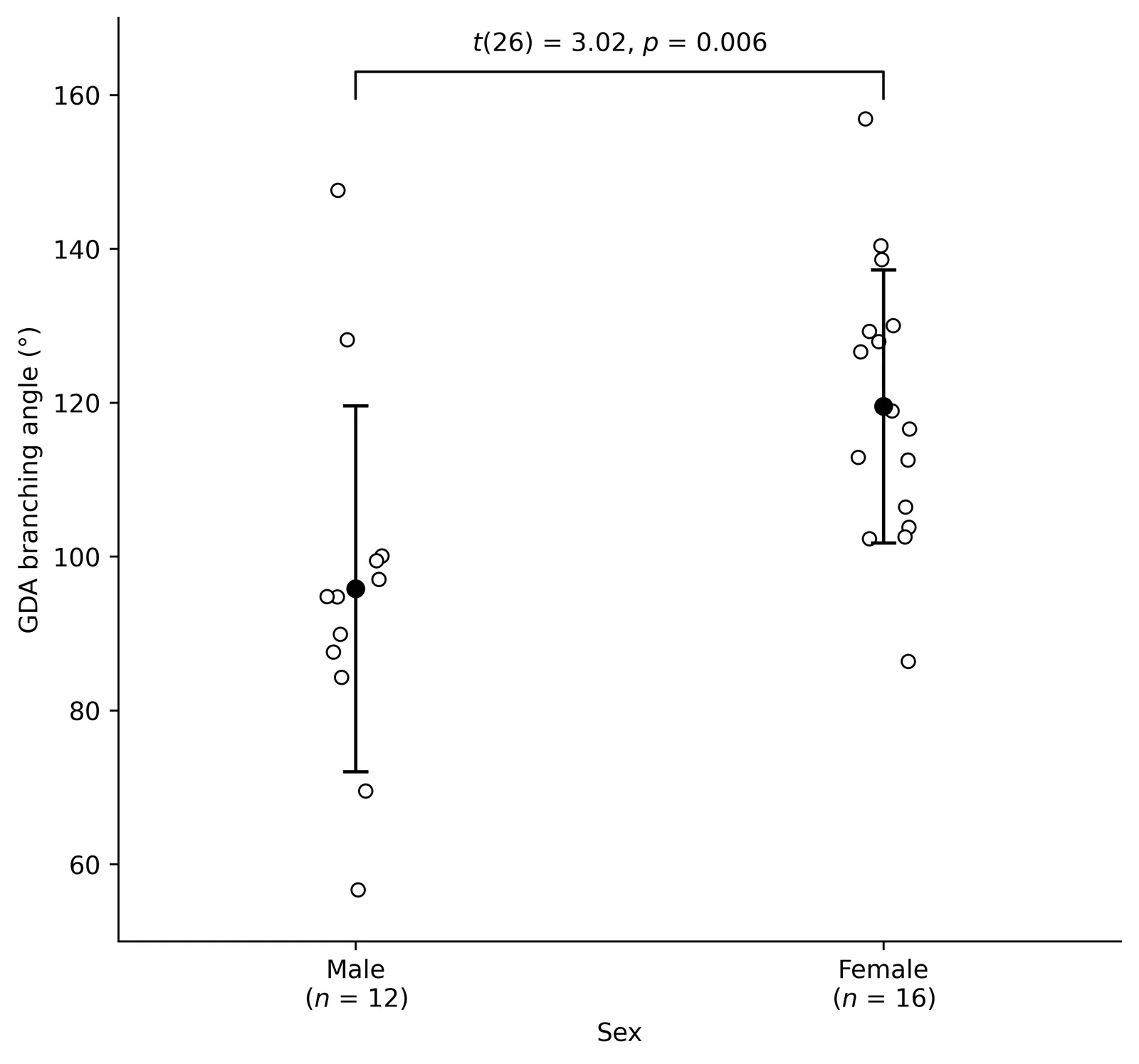
GDA branching angle according to donor sex Individual open circles represent donor measurements; filled circles and error bars represent mean ± standard deviation. Female donors demonstrated a significantly larger mean GDA branching angle than male donors (independent-samples Student’s *t*-test, *t*(26) = 3.02, *p* = 0.006). GDA: gastroduodenal artery

**Table 2:** Sex-based differences in gastroduodenal artery (GDA) morphometric measurements Values are presented as mean ± standard deviation (SD). Mean differences are calculated as male − female. Branching angle was compared using independent-samples Student’s *t*-test. Diameter was compared using Welch’s independent-samples *t*-test because the assumption of homogeneity of variance was violated. Cohen’s *d* was calculated using the pooled SD. CI: confidence interval

Measurement	Male, mean ± SD	Female, mean ± SD	Mean difference (male − female)	95% CI for mean difference	Test statistic	*p*-value	Cohen’s *d*
Branching angle (°)	95.83 ± 23.79	119.51 ± 17.75	−23.68°	−39.79 to −7.58	*t*(26) = −3.02	0.006	1.15
Diameter (mm)	5.67 ± 2.57	3.17 ± 0.70	2.49 mm	0.84 to 4.15	Welch’s *t*(12.23) = 3.27	0.007	1.42

The difference was statistically significant (*t*(26) = 3.02, *p* = 0.006), with a large effect size (Cohen’s *d* = 1.15). The mean between-group difference (female-male) was 23.68° (95% CI, 7.58°-39.79°).

Diameter

Mean GDA diameter was larger in male donors than in female donors (5.67 ± 2.57 mm vs. 3.17 ± 0.70 mm; Figure 4). Diameters ranged from 3.42 mm to 11.06 mm in male donors and from 1.73 mm to 4.57 mm in female donors.

**Figure 4:**
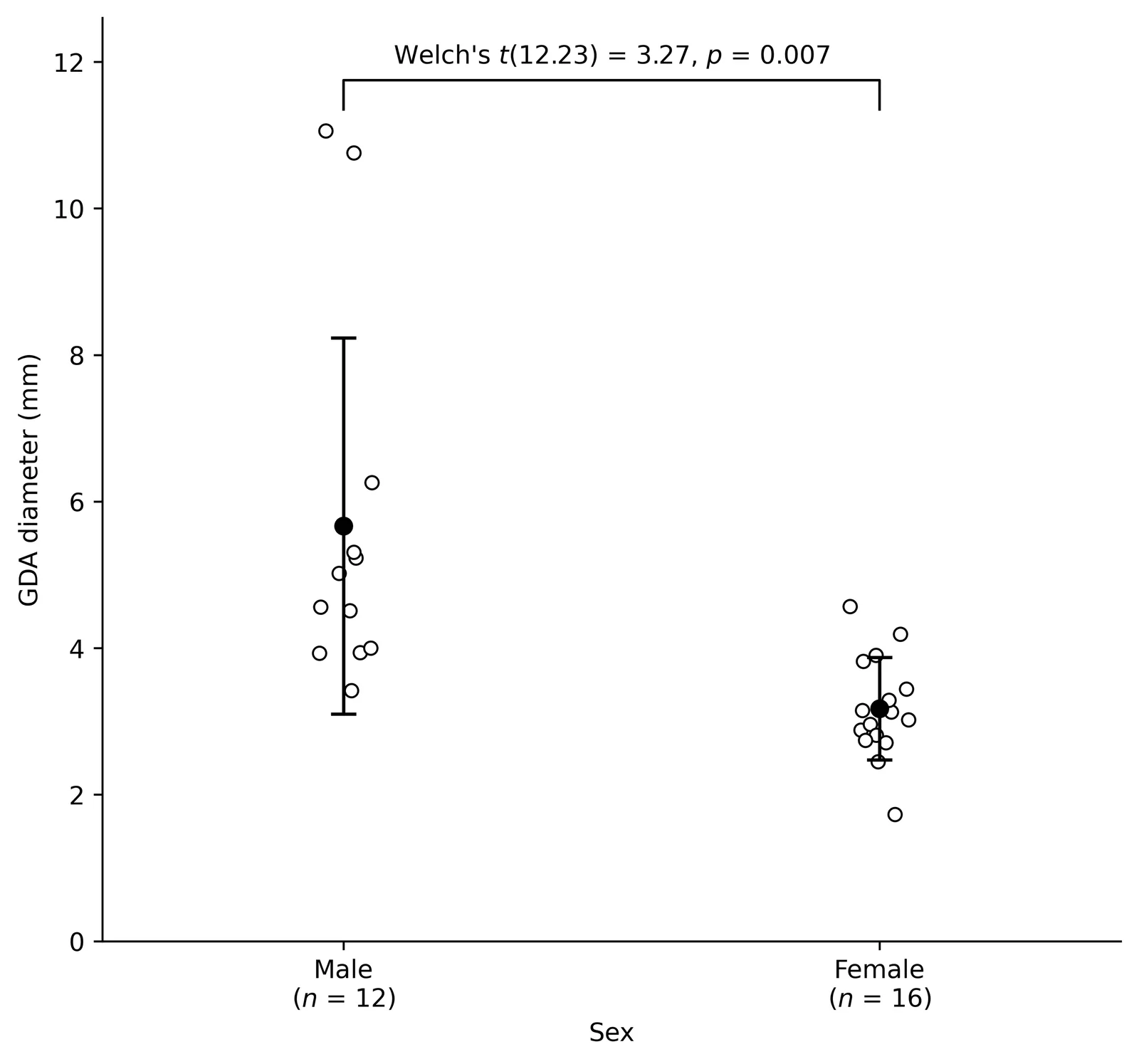
GDA diameter according to donor sex Individual open circles represent donor measurements; filled circles and error bars represent mean ± standard deviation. Male donors demonstrated a significantly larger mean GDA diameter than female donors (Welch’s independent-samples *t*-test, *t*(12.23) = 3.27, *p* = 0.007). GDA: gastroduodenal artery

The difference was statistically significant (Welch’s *t*(12.23) = 3.27, *p* = 0.007) with a large effect size (Cohen’s *d* = 1.42). The mean between-group difference (male-female) was 2.49 mm (95% CI, 0.84 mm-4.15 mm). A Mann-Whitney U test, performed as a sensitivity analysis because of departure from normality, similarly demonstrated a significant between-group difference (*p* < 0.001).

## Discussion

Sex-based differences in GDA geometry were identified, with male donors demonstrating larger diameters and smaller branching angles than female donors. These findings demonstrate sex-associated variation in GDA morphology. Although clinical imaging and procedural outcomes were not evaluated in this study, the observed anatomical differences may have relevance to endovascular planning and CT angiography interpretation.

Previous studies described normal GDA diameters of approximately 4-6 mm and emphasized the role of the GDA in collateral circulation, particularly in the setting of celiac artery stenosis [1,17]. In the present study, a subset of donors demonstrated enlarged GDA diameters without evidence of associated pathology, consistent with prior observations that increased caliber may reflect compensatory flow [18]. Previous computational and experimental studies also demonstrated that arterial geometry influences hemodynamic parameters, including wall shear stress and pressure distribution, with smaller branching angles associated with increased hemodynamic stress [2,12-14]. In addition to hemodynamic effects, acute branching angles have been associated with technical catheter navigation during endovascular procedures, including microcatheter access in angled vascular segments [19]. Accordingly, the sex-based differences in diameter and branching angle observed in this study could represent one source of anatomical variation relevant to endovascular access, although procedural difficulty was not directly assessed.

The observed differences may also be relevant when interpreting normal anatomical variation on CT angiography during preprocedural vascular assessment. Previous studies have demonstrated the utility of CT angiography-based vascular morphometry for anatomical characterization and preprocedural assessment, where differences in vascular anatomy may influence endovascular strategy [20]. However, the present study did not evaluate CT angiographic measurements or diagnostic performance, and the applicability of these cadaveric findings to clinical imaging requires further validation. Similarly, the observed variation in baseline GDA diameter suggests that uniform diagnostic thresholds may not fully reflect underlying anatomical variability, although this possibility requires larger in vivo cohorts.

This study has several limitations. First, the use of formalin-embalmed cadaveric donors may introduce postmortem and fixation-related changes affecting vessel diameter and elasticity compared with in vivo conditions. Cadaveric morphometric analysis nevertheless permits direct vessel visualization and controlled measurement conditions that are difficult to achieve consistently using clinical imaging datasets. Second, limited clinical information precluded assessment of potential confounding factors, including body size, vascular comorbidities, prior interventions, and hemodynamic factors that may influence vascular morphology. Third, measurements were derived from two-dimensional images and may not fully capture three-dimensional vessel geometry, particularly when vessels deviated from the imaging plane. Although measurements were independently repeated by two blinded observers, formal interobserver and intraobserver reliability statistics were not calculated, limiting quantitative assessment of measurement reproducibility. The exclusion of donors with damaged vessels or anatomy altered by prior regional surgery may also have introduced selection bias, as the final sample may not fully represent the anatomical variability of the original donor population. Finally, the relatively small sample size and absence of anatomical variants limit generalizability. Future studies incorporating larger cohorts and in vivo imaging, particularly contrast-enhanced CT angiography, are needed to further characterize sex-based variation in GDA anatomy.

## Conclusions

Sex-based differences in GDA geometry were identified in this cadaveric cohort, with male donors exhibiting significantly larger vessel diameters and smaller branching angles than female donors. These findings demonstrate measurable sex-associated morphologic variation within the GDA and may have relevance to vascular appearance on angiographic imaging and technical considerations during endovascular procedures.

Recognition of these anatomical differences may be relevant to CT angiography interpretation and procedural planning in interventional radiology and hepatopancreatobiliary surgery; however, these potential clinical applications were not directly evaluated in the present study. Given the cadaveric design and limited sample size, larger studies incorporating in vivo imaging and clinical outcomes are needed to validate these findings and determine their clinical significance. The present findings provide quantitative data that may help define the range of normal anatomical variation and support future investigations of sex-specific vascular morphology.
